# A Novel Monthly Dosing Regimen of Risedronate for the Treatment of Postmenopausal Osteoporosis: 2-Year Data

**DOI:** 10.1007/s00223-012-9668-4

**Published:** 2012-11-13

**Authors:** Michael R. McClung, Claude-Laurent Benhamou, Zulema Man, Witold Tlustochowicz, Jose R. Zanchetta, Rachelle Eusebio, Ana M. Balske, Ellen Matzkin, Wojciech P. Olszynski, Robert Recker, Pierre D. Delmas

**Affiliations:** 1 Oregon Osteoporosis Center, 5050 NE Hoyt Street, Suite 626, Portland, OR 97213 USA; 2 EA 4708 Orléans University and Orléans Hospital, Orléans, France; 3 Centro Médico TIEMPO, Buenos Aires, Argentina; 4 Klinika Chorob Wewnetrznych i Reumatologii, Centralny Szpital Kliniczny MON Wojskowy Instytut Medyczny, Warsaw, Poland; 5 Instituto de Investigaciones Metabolicas, Buenos Aires, Argentina; 6 Procter and Gamble Company, Mason, OH USA; 7 Abbott Laboratories, Abbott Park, Chicago, IL USA; 8 The sanofi-aventis Group, Bridgewater, NJ USA; 9 Saskatoon Osteoporosis Center, Sasketoon, SK Canada; 10 Osteoporosis Research Center, Creighton University, Omaha, NE USA; 11 INSERM Research Unit 831, University of Lyon, Lyon, France

**Keywords:** Bone mineral density, Bone turnover markers, Osteoporosis, Risedronate

## Abstract

This 2-year trial evaluated the efficacy and tolerability of a monthly oral regimen of risedronate. Postmenopausal women with osteoporosis were randomly assigned to double-blind treatment with risedronate 75 mg on 2 consecutive days each month (2CDM) or 5 mg daily. The primary end point was the percentage change from baseline in lumbar spine bone mineral density (BMD) at 12 months. Secondary end points included the change in BMD of the lumbar spine and proximal femur and in bone turnover markers as well as the number of subjects with at least one new vertebral fracture over 24 months. Among 1,229 patients who were randomized and received at least one dose of risedronate, lumbar spine BMD was increased in both treatment groups: mean percentage change from baseline was 4.2 ± 0.19 and 4.3 ± 0.19 % in the 75 mg 2CDM and 5 mg daily groups, respectively, at month 24. The treatment difference was 0.17 (95 % confidence interval −0.35 to 0.68). There were no statistically significant differences between treatment groups on any secondary efficacy parameters. Both treatment regimens were well tolerated. Risedronate 75 mg 2CDM was noninferior in BMD efficacy and did not show a difference in tolerability compared to 5 mg daily after 24 months of treatment in women with postmenopausal osteoporosis. This monthly regimen may provide a more convenient dosing schedule to some patients with postmenopausal osteoporosis.

## Introduction

Oral bisphosphonates are the most commonly prescribed drugs for the treatment of postmenopausal osteoporosis. Each of the three available oral bisphosphonates was originally developed for daily dosing. However, because the drugs have to be given while fasting, with a 30–60 min interval before the patient may eat, drink beverages other than water, or take other medicines, many patients found daily dosing to be inconvenient. Bisphosphonates bind with variable affinity to bone mineral and reside within the bone matrix for long periods after dosing. The drugs remain active on the surface of bone, providing the opportunity to develop a range of dosing schedules. When given the option of daily dosing or less frequent (weekly or monthly) dosing, most patients chose the latter [1]. Adherence to therapy is modestly improved with weekly or monthly dosing regimens compared with daily dosing [2, 3].

Daily dosing with risedronate, a potent nitrogen-containing bisphosphonate, has been found to reduce the incidence of vertebral, nonvertebral, and hip fracture [4–6]. It was later demonstrated that 35 mg once a week provided similar efficacy, assessed by changes in bone mineral density (BMD), and safety to the daily regimen [7]. Risedronate 75 mg each day for 2 consecutive days a month (2CDM) has also been shown to be an effective treatment regimen for postmenopausal osteoporosis [8]. In that article, the efficacy and safety of risedronate 75 mg 2CDM was compared with the 5 mg daily regimen in postmenopausal women with osteoporosis over a 12-month period. Risedronate 75 mg 2CDM was shown to be noninferior in BMD response and similar in tolerability to the 5 mg daily dosing regimen after 12 months [8]. This report provides the 2-year data from this study and assesses whether 2CDM provides continued efficacy and safety similar to the 5 mg daily regimen in postmenopausal women with osteoporosis during the second year of treatment.

## Methods

### Study Design

Details of the study design, patient population, and inclusion criteria have previously been reported [8]. Briefly, this was a multicenter, double-blind, randomized, active-controlled, parallel-group, noninferiority study designed to compare two oral dosing regimens of risedronate for the treatment of postmenopausal osteoporosis. The trial was conducted in accordance with the guidelines of the International Conference on Harmonization for Good Clinical Practice, and the study protocol was approved by Independent Ethics Committees at each participating study center. The study’s ClinicalTrials.gov identifier was NCT00358176.

### Subjects

Healthy, ambulatory women who were at least 50 years old and had been postmenopausal for 5 years or more were eligible for inclusion in this study if they had osteoporosis as defined by a lumbar spine T-score of −2.5 or lower or a T-score of −2.0 or lower and at least one prevalent vertebral fracture. Subjects were excluded if they had received any bone active drugs within 3 months of the first dose of the study medication, had a body mass index of >32 kg/m^2^, or had a history of drug or alcohol abuse.

### Treatment

Subjects were randomly assigned in a 1:1 ratio to one of two risedronate oral dosing regimens: 75 mg 2CDM or 5 mg daily. Subjects were told to take their medication with water, in an upright position, on an empty stomach in the morning, at least 30 minutes before their first food or drink of the day. Supplementation with 1,000 mg of elemental calcium and 400–800 IU of vitamin D was permitted, depending on supplement availability and customary local practice. Patient compliance with the assigned treatment protocol was determined by tablet counts at every visit.

### Outcome Measures

Bone mineral density (BMD) of the lumbar spine and total hip was measured by dual energy X-ray absorptiometry (DXA) at baseline and months 6, 12, and 24. All DXA scans were performed using Lunar (General Electric, Madison, WI, USA) and Hologic (Hologic Inc., Waltham, MA, USA) machines with all scans for each subject being acquired on the same machine. SYNARC (SYNARC, San Francisco, CA, USA) performed all DXA analyses and ensured DXA equipment stability throughout the study. Lateral X-rays of the thoracic and lumbar spine were obtained at baseline and at month 24 or at early termination visit. All X-rays were analyzed at the SYNARC central reading facility using a semiquantitative scoring of digitized films. Fasting samples were collected for bone turnover markers (BTMs) including urinary *N*-telopeptide (uNTx) and serum bone-specific alkaline phosphatase (sBAP).

The primary efficacy end point was the mean percentage change from baseline in lumbar spine BMD at month 12. Secondary efficacy measures included the mean percentage change from baseline in lumbar spine BMD at month 24 and end point; mean percentage change from baseline in total hip, femoral neck, and trochanter BMD at month 24 and end point; mean percentage change in uNTx and sBAP at month 24 and end point; the number of subjects with ≥1 new vertebral fracture at month 24 and end point. Month 24 values include only assessments made when subjects returned for the study visit after completing 24 months of therapy. End point values included the 24-month values plus the last results of other subjects for whom 24-month values were not available but who had undergone at least one BMD, bone turnover marker, or spine X-ray assessment performed after taking at least one dose of study drug.

### Safety

A physical examination was performed before treatment and at months 12 and 24. Vital signs and adverse events were assessed and recorded at all scheduled visits. Serum chemistries, including calcium and liver function tests and hematology tests were performed at 6 month intervals and urinalysis was performed annually.

### Statistical Analyses

The primary efficacy analysis was a test of noninferiority comparing the mean percentage change from baseline in lumbar spine BMD in the 75 mg 2CDM and 5 mg daily groups after 12 months. Noninferiority was to be declared if the upper bound of the 2-sided 95 % confidence interval (95 % CI) for the treatment difference (5 mg daily minus 75 mg 2CDM) did not exceed the predefined noninferiority margin of 1.5 %. This margin was based on the mean difference in BMD percentage change between 5 mg once daily and placebo [4, 5]. The primary analysis population was all subjects who were randomized, received at least one dose of study drug, and had evaluable measurements of lumbar spine BMD at both baseline and month 12. The noninferiority analysis was performed in a similar manner after 24 months. If the upper limit of the 95 % 2-sided confidence interval for the treatment difference obtained from the ANOVA model did not exceed the predefined noninferiority margin of 2.0 %, then the 75 mg 2CDM regimen would be declared noninferior to the 5 mg daily regimen at month 24. Investigative centers were pooled by geographic region. An analysis of variance (ANOVA) was performed with treatment and pooled centers as fixed effects and percentage change from baseline in lumbar spine BMD as the response variable. Continuous secondary efficacy variables were analyzed using similar ANOVA methods. Two-sided 95 % CIs were constructed for changes from baseline, between treatment groups, and within treatment groups. No statistical test for superiority of the 2CDM dose to the daily dose of risedronate was performed.

## Results

### Subjects

**Fig. 1:**
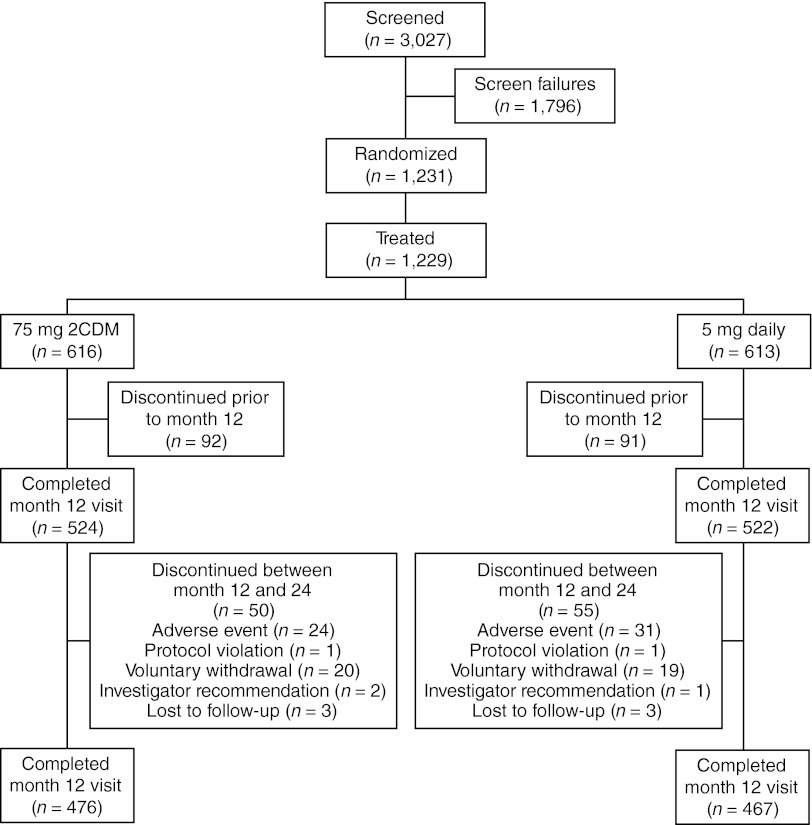
Subject disposition. *2CDM* two consecutive days each month. One subject in each of the treatment groups missed their month 12 visit but continued on to year 2. These two subjects were not counted in month 12 visit but were included in the month 24 visit

As reported previously [8], a total of 3,027 women were screened at 61 sites in 11 countries, with 1,231 subjects enrolled and randomized for treatment. At least one dose of risedronate was received by 1,229 subjects. Figure 1 illustrates subject disposition throughout the study period.

Demographics of the subjects in each treatment group were similar between the two treatment groups (Table 1); details have previously been reported [8]. Mean age was approximately 65 years, and mean lumbar spine and total hip T-scores were approximately −3.2 and −1.9, respectively. Approximately 30 % of subjects had at least one prevalent fracture.

**Table 1:** Subject demographics at baseline

Characteristic	75 mg 2CDM (*n* = 616)	5 mg daily (*n* = 613)
Age (years), mean ± SD	65.1 ± 7.80	64.2 ± 7.75
Lumbar spine BMD T-score, mean ± SD	−3.16 ± 0.54	−3.17 ± 0.56
Total hip BMD T-score, mean ± SD	−1.91 ± 0.77	−1.86 ± 0.78
uNTX/creatinine (nmol BCE/nmol), mean ± SD	60.8 ± 39.9	59.0 ± 35.7
sBAP (μg/ml), mean ± SD	15.13 ± 5.15	15.01 ± 5.37

*BMD* bone mineral density, *2CDM* two consecutive days each month, *sBAP* serum bone-specific alkaline phosphatase, *uNTX* urinary N-telopeptide

Similar proportions of subjects in each study group completed the 24-month study: 476 (77 %) in the 75 mg 2CDM group and 467 (76 %) in the 5 mg daily group. Mean treatment duration with risedronate was similar for both treatment groups: 625 days for subjects receiving 75 mg 2CDM, and 627 days for subjects receiving 5 mg daily. About 15 % of subjects had withdrawn from the study by 12 months. The reasons for treatment discontinuation over 24 months are summarized in Table 2. The proportion of subjects with over 80 % compliance was 96 % for the 75 mg 2CDM group, and 97 % for the 5 mg daily group.

**Table 2:** Subject withdrawal by month 24 and reasons for treatment discontinuation

Reason	75 mg 2CDM, *n* (%) (*n* = 616)	5 mg daily, *n* (%) (*n* = 613)
Discontinued before month 24	142 (23.0)	146 (23.8)
Adverse event	80 (13.0)	86 (14.0)
Protocol violation	6 (1.0)	2 (0.3)
Voluntary withdrawal	43 (7.0)	45 (7.3)
Investigator recommendation	7 (1.1)	7 (1.1)
Lost to follow-up	6 (1.0)	6 (1.0)

*2CDM* two consecutive days each month

### Primary Efficacy

**Fig. 2:**
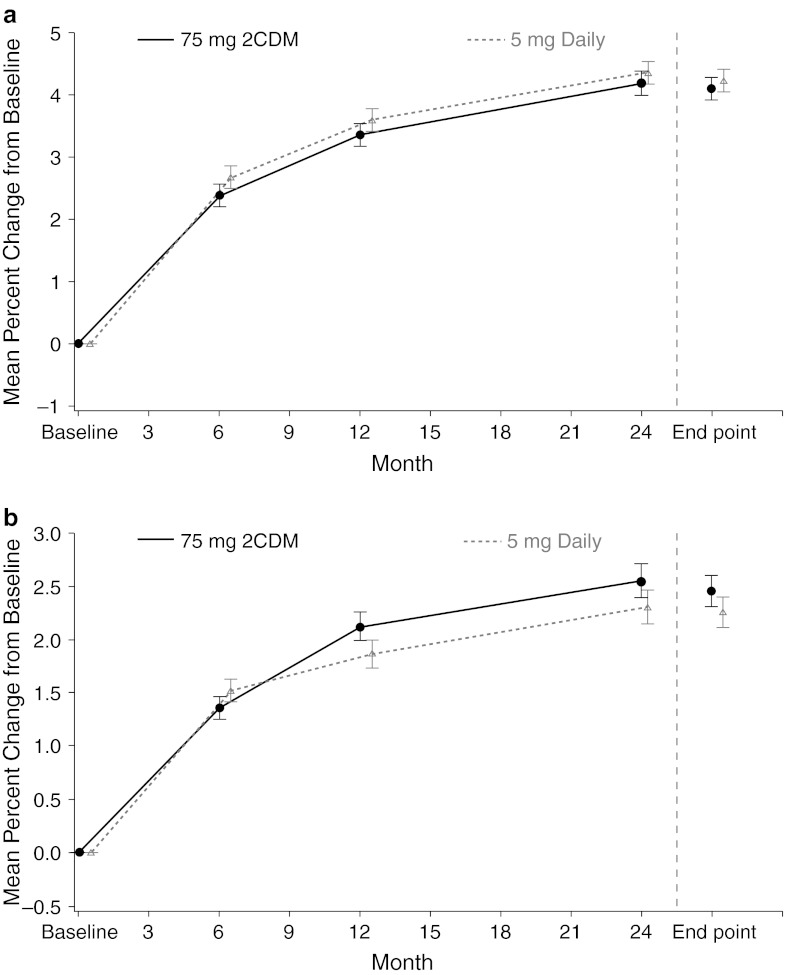
LS mean (±SE) percentage change from baseline in **a** lumbar spine and **b** total hip BMD by visit. *2CDM* two consecutive days each month*, BMD* bone mineral density, *LS* least squares

As reported previously [8], both risedronate 75 mg 2CDM and 5 mg daily increased lumbar spine BMD at month 12, and the results indicate that the 75 mg 2CDM dosing regimen was noninferior to the 5 mg daily dosing regimen (treatment difference 0.21; 95 % CI −0.19 to 0.62). Consistent with these data, lumbar spine BMD at month 24 was increased in both treatment groups: 4.2 ± 0.19 and 4.3 ± 0.19 % in the 75 mg 2CDM and 5 mg daily groups, respectively, and at end point: 4.1 ± 0.18 and 4.2 ± 0.18 %, respectively (Fig. 2). The lumbar spine mean difference between treatment groups was 0.17 (95 % CI −0.35 to 0.68) at months 24 and 0.13 (95 % CI −0.34 to 0.60) at end point. The upper bound of the 95 % CI was less than the predefined noninferiority margin of 2.0 %, demonstrating the 2CDM regimen to be noninferior to the daily regimen at 24 months and at end point.

### Secondary Efficacy

**Fig. 3:**
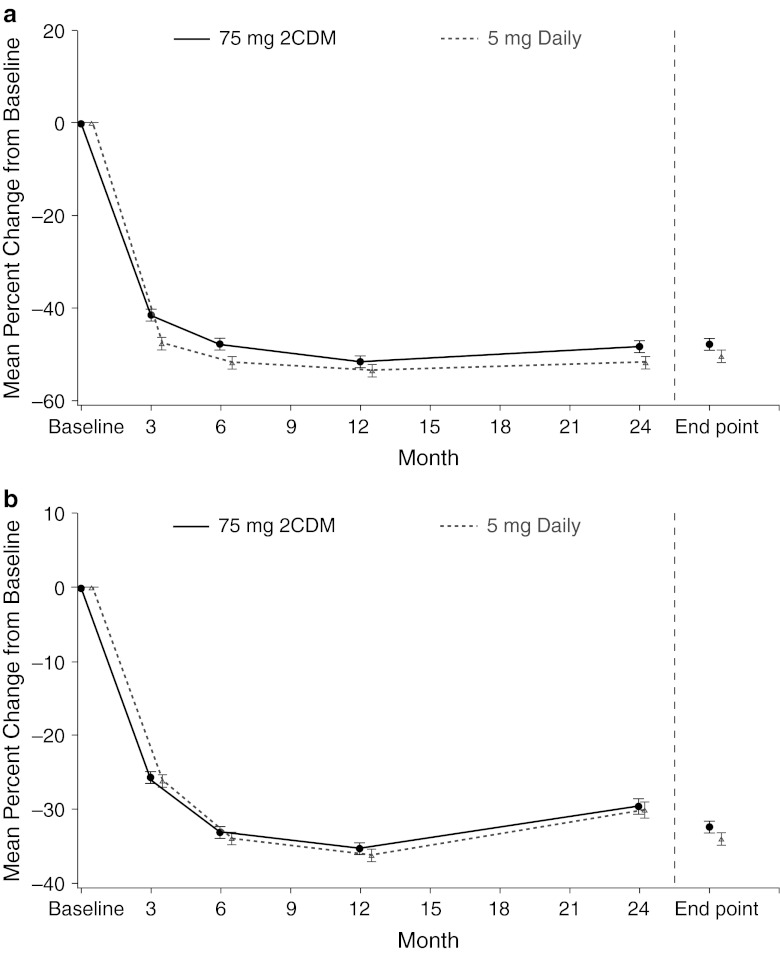
LS mean (±SE) percentage change from baseline in **a** uNTX/creatinine and **b** sBAP. *2CDM* two consecutive days each month*, sBAP* serum bone-specific alkaline phosphatase, *uNTX* urinary N-telopeptide

There were no statistically significant differences between treatment groups for any of the secondary efficacy parameters at month 24 or end point, which included change from baseline in total hip (Fig. 2), femoral neck, and trochanter BMD (Table 3), and uNTx and sBAP (Fig. 3). At end point, 16 (2.9 %) patients treated with 75 mg 2CDM and 15 (2.7 %) patients treated with 5 mg daily had experienced 1 or more new morphometric vertebral fractures.

**Table 3:** Mean percentage change from baseline in BMD in the ITT population

Characteristic	75 mg 2CDM		5 mg daily		5 mg daily—75 mg 2CDM, LS mean difference (95 % CI)^a^
	*n*	LS mean	*n*	LS mean	
Lumbar spine BMD					
Baseline (g/cm^2^)	615	0.742	613	0.744	
Percentage change from baseline					
Month 6	544	2.385*	549	2.672*	0.287 (−0.089; 0.662)
Month 12	527	3.361*	531	3.588*	0.227 (−0.176; 0.630)
Month 24	479	4.181*	474	4.348*	0.167 (−0.345; 0.679)
End point	553	4.102*	552	4.232*	0.130 (−0.341; 0.600)
Total hip BMD					
Baseline (g/cm^2^)	600	0.734	601	0.741	
Percentage change from baseline					
Month 6	542	1.364*	551	1.522*	0.158 (−0.141 to 0.457)
Month 12	516	2.121*	522	1.862*	−0.259 (−0.603 to 0.085)
Month 24	468	2.549*	462	2.307*	−0.243 (−0.657 to 0.171)
End point	553	2.457*	558	2.255*	−0.202 (−0.577 to 0.173)
Femoral neck BMD					
Baseline (g/cm^2^)	600	0.665	601	0.670	
Percentage change from baseline					
Month 6	542	1.039*	551	0.800*	−0.238 (−0.603 to 0.126)
Month 12	516	1.615*	522	1.145*	−0.470 (−0.894 to −0.047)
Month 24	468	1.981*	462	1.677*	−0.304 (−0.852 to 0.243)
End point	553	1.948*	558	1.576*	−0.372 (−0.864 to 0.121)
Femoral trochanter BMD					
Baseline (g/cm^2^)	600	0.570	601	0.573	
Percentage change from baseline					
Month 6	542	2.081*	551	2.424*	0.344 (−0.150 to 0.837)
Month 12	516	2.971*	522	3.022*	0.051 (−0.494 to 0.595)
Month 24	468	3.957*	462	3.870*	−0.087 (−0.741 to 0.567)
End point	553	3.809*	558	3.796*	−0.013 (−0.603 to 0.577)

Consists of ITT subjects with analyzable baseline and postbaseline data for the relevant visit

*ANOVA* analysis of variance, *BMD* bone mineral density, *2CDM* two consecutive days each month, *CI* confidence interval (2-sided), *ITT* intention to treat, *LS* least squares, *n* number of subjects in the indicated population with values at baseline and the relevant visit

* Statistically significant difference from baseline determined from a 95 % CI unadjusted for multiple comparisons

^a^Adjusted means, mean differences, and confidence intervals are from an ANOVA model containing treatment and pooled investigative center

### Safety

After 24 months, subjects in both treatment groups experienced comparable percentages of treatment-emergent adverse events (TEAEs) (Table 4). The overall proportion of subjects who withdrew from the study as a result of TEAEs was similar in the two treatment groups, comprising 12.8 % of the subjects in the 75 mg 2CDM group and 13.9 % in the 5 mg daily group. Nine deaths occurred during the 24 months of the study: three in the 75 mg 2CDM group and six in the 5 mg daily group. None of the deaths were considered to be treatment-related. A comparable proportion of subjects in both groups experienced musculoskeletal TEAEs and upper gastrointestinal TEAEs. No reports of fever or influenza-like illness, potentially representing acute phase reactions, occurred in year 2 of the study. No case of atypical femoral fracture or osteonecrosis of the jaw (ONJ) was identified in our study.

**Table 4:** Overview of TEAEs (safety population)

Characteristic	75 mg 2CDM, *n* (%) (*n* = 616)	5 mg daily, *n* (%) (*n* = 613)
Subjects with TEAEs	561 (91.1)	551 (89.9)
Subjects with serious TEAEs	89 (14.4)	66 (10.8)
Subjects withdrawn as a result of a TEAE	79 (12.8)	85 (13.9)
Subjects with TEAEs resulting in death	3 (0.5)	6 (1.0)
AEs of special interest		
Subjects with upper GI TEAEs	162 (26.3)	169 (27.6)
Subjects with moderate to severe upper GI TEAEs	59 (9.6)	53 (8.6)
Subjects with acute phase reaction^a^	4 (0.7)	0
Subjects with ≥1 morphometric vertebral fracture	16 (2.9)	15 (2.7)
Subjects with clinical vertebral fracture	6 (1.0)	5 (0.8)
Subjects with vertebral clinical fracture TEAEs	6 (1.0)	5 (0.8)
Subjects with nonvertebral clinical fracture TEAEs	35 (5.7)	31 (5.1)
Subjects with osteoporosis-related fractures TEAEs^b^	21 (3.4)	13 (2.1)
Subjects with selected musculoskeletal TEAEs	190 (30.8)	187 (30.5)

Safety population includes subjects who were randomized to the treatment groups and received ≥1 documented dose of investigational product

*AE* adverse event, *2CDM* two consecutive days each month, *GI* gastrointestinal, *ITT* intent to treat, *TEAE* treatment-emergent adverse event

^a^Includes fever and influenza-like illness during first 5 days of treatment

^b^Includes fractures at wrist, hip, leg, clavicle, humerus, and pelvis

The incidence of clinical vertebral and nonvertebral fracture TEAEs were similar in the two treatment groups (Table 4). It should be noted, however, that this study was not statistically powered to detect differences in fracture rates as efficacy outcome measures.

## Discussion

Although a 5 mg daily risedronate dosing regimen was developed initially, less frequent dosing regimens with similar efficacy and safety profiles are now available. This study demonstrated that risedronate 75 mg 2CDM is noninferior to the 5 mg daily regimen in postmenopausal women with osteoporosis after 24 months of treatment. These data are consistent with the noninferiority of the 2CDM dose compared to the daily dosing regimen reported in the interim analysis after 12 months of therapy [8]. The smaller increase in BMD during the second year of therapy (0.8 %) compared with the 3.4 % observed during the first year of treatment is consistent with previous risedronate studies [4, 5]. This pattern of BMD response is typical of that observed with all antiresorptive agents and does not connote loss of efficacy after the first year. Additionally, comparison of the BMD response at the end of the first and second years of treatment is complicated because not all subjects included in the month 12 results continued to be included in the month 24 analysis. Secondary efficacy analyses also showed no differences between the 75 mg 2CDM and the 5 mg daily regimens with respect to BMD at the proximal femur or in bone turnover markers.

Overall, the safety profile and tolerability of risedronate 75 mg 2CDM over 24 months of therapy was similar to that of the 5 mg daily regimen. In particular, comparable percentages of musculoskeletal and upper gastrointestinal TEAEs were found across treatment groups. Although infrequent mild or moderate acute phase reactions were observed at the beginning of therapy in the 75 mg 2CDM group [8], there was no reported incidence of these symptoms during year 2. This is similar to the pattern of acute phase reactions that occurs with intravenous bisphosphonates where symptoms primarily occur after the initial dose [9].

These data are consistent with previous studies that have demonstrated favorable tolerability and safety profiles with risedronate, independent of dosing regimen [4, 5, 7, 10–14]. Additionally, in clinical trials, long-term treatment with risedronate does not increase the incidence of adverse events, including upper gastrointestinal complaints [15, 16]. Concern exists about skeletal safety with long term bisphosphonate therapy. No case of ONJ or atypical femoral fractures has been identified in our study or any of the Phase 3 risedronate clinical trials that comprise more than 25,000 patient-years of exposure. However, fewer than 200 patients were followed for more than 5 years [16].

The noninferiority of the 75 mg 2CDM dose demonstrated at month 24 are consistent with the results of the 1-year interim report [8]. These are not unexpected findings because the 75 mg 2CDM dosing regimen represents the same cumulative monthly dose of risedronate (150 mg) as the 5 mg daily dosing regimen, and a previous study showed that plasma blood levels and pharmacokinetic parameters are linear between the 5 mg daily dose and the 75 mg 2CDM [17]. Additionally, the response to risedronate given as a single dose of 150 mg monthly is similar to that of the 5 mg daily dose [11]. Although no direct comparison has been made between the 75 mg 2CDM dose and the 150 mg once monthly dose of risedronate, the clinical responses with the 2CDM regimen at month 24 are similar to the responses observed with risedronate 150 mg given once monthly as a single tablet. After 2 years, the mean increase in lumbar spine BMD from baseline was 4.2 % with the 2CDM dose. That same metric was 3.9 % with the 5 mg daily dose and 4.2 % with the 150 mg once monthly dose in a previous study [18].

A limitation of this study is that the primary efficacy measure was BMD rather than vertebral and nonvertebral fracture risk. However, the occurrence of new vertebral fractures was a secondary efficacy measure, and there was no difference between groups in this parameter at 12 and 24 months. Relative to the historical placebo group, the risk of vertebral fracture at month 12 with the 75 mg 2CDM dose of risedronate was reduced by 79 % (5.1 % in historical placebo group vs. 1.1 % in the risedronate 75 mg 2CDM group; relative risk = 0.21; 95 % CI 0.05 to 0.88; *p* = 0.016) [19]. The US Food and Drug Administration (FDA) and the European Agency for the Evaluation of Medicinal Products guidelines have determined that alternate dose forms can be approved based upon BMD for a bisphosphonate that has established fracture risk reduction [20].

Preference among osteoporosis treatment options is mainly influenced by fracture efficacy, tolerability, and convenience of the dosing regimen [21, 22]. As has been demonstrated with once weekly and once monthly dosing forms of risedronate, risedronate 75 mg 2CDM has an efficacy and safety profile similar to the 5 mg daily regimen over 2 years [10, 18]. The once-a-month dose is available in some, but not all, countries. For patients without access to once-a-month risedronate, the 2CDM treatment regimen offers an additional treatment option for women with postmenopausal osteoporosis who prefer a monthly dosing regimen of a drug proven to reduce the risk of vertebral and nonvertebral fractures.

In conclusion, risedronate 75 mg 2CDM is noninferior to the 5 mg daily dosing regimen after 2 years of treatment in women with postmenopausal osteoporosis. This dosing regimen is as safe and well tolerated as the 5 mg daily dosing regimen. Risedronate 75 mg 2CDM provides a more convenient dosing schedule with a bisphosphonate that has proven fracture reduction efficacy at both vertebral and nonvertebral sites, including those of the hip [4, 6].
